# Thrombin Receptor-Activating Protein (TRAP)-Activated Akt Is Involved in the Release of Phosphorylated-HSP27 (HSPB1) from Platelets in DM Patients

**DOI:** 10.3390/ijms17050737

**Published:** 2016-05-14

**Authors:** Haruhiko Tokuda, Gen Kuroyanagi, Masanori Tsujimoto, Rie Matsushima-Nishiwaki, Shigeru Akamatsu, Yukiko Enomoto, Hiroki Iida, Takanobu Otsuka, Shinji Ogura, Toru Iwama, Kumi Kojima, Osamu Kozawa

**Affiliations:** 1 Department of Clinical Laboratory, National Center for Geriatrics and Gerontology, 7-430 Morioka, Obu, Aichi 474-8511, Japan; koji-kun@ncgg.go.jp; 2 Department of Pharmacology, Gifu University Graduate School of Medicine, 1-1 Yanagido, Gifu 501-1194, Japan; kokuryugen@yahoo.co.jp (G.K.); masanori-t.117@hotmail.co.jp (M.T.); riemn@gifu-u.ac.jp (R.M.-N.); okozawa@gifu-u.ac.jp (O.K.); 3 Department of Orthopedic Surgery, Nagoya City University Graduate School of Medical Sciences, 1-Kawasumi, Nagoya 467-8601, Japan; t.otsuka@med.nagoya-cu.ac.jp; 4 Department of Neurosurgery, Gifu University Graduate School of Medicine, 1-1 Yanagido, Gifu 501-1194, Japan; enomoto@gifu-u.ac.jp (Y.E.); tiwama@gifu-u.ac.jp (T.I.); 5 Department of Anesthesiology and Critical Care Medicine, Chuno Kosei Hospital, 5-1 Wakakusa, Seki, Gifu 501-6062, Japan; shigeruakamatsu@yahoo.co.jp; 6 Department of Anesthesiology and Pain Medicine, Gifu University Graduate School of Medicine, 1-1 Yanagido, Gifu 501-1194, Japan; iida@gifu-u.ac.jp; 7 Department of Emergency and Disaster Medicine, Gifu University Graduate School of Medicine, 1-1 Yanagido, Gifu 501-1194, Japan; oguras@gifu-u.ac.jp

**Keywords:** thrombin-activating protein, HSP27, Akt, platelet, diabetes mellitus

## Abstract

It is generally known that heat shock protein 27 (HSP27) is phosphorylated through p38 mitogen-activated protein (MAP) kinase. We have previously reported that HSP27 is released from human platelets associated with collagen-induced phosphorylation. In the present study, we conducted an investigation into the effect of thrombin receptor-activating protein (TRAP) on the release of HSP27 in platelets in type 2 diabetes mellitus (DM) patients. The phosphorylated-HSP27 levels induced by TRAP were directly proportional to the aggregation of platelets. The levels of phosphorylated-HSP27 (Ser-78) were correlated with the levels of phosphorylated-p38 MAP kinase and phosphorylated-Akt in the platelets stimulated by 10 µM TRAP but not with those of phosphorylated-p44/p42 MAP kinase. The levels of HSP27 released from the TRAP (10 µM)-stimulated platelets were correlated with the levels of phosphorylated-HSP27 in the platelets. The released platelet-derived growth factor-AB (PDGF-AB) levels were in parallel with the HSP27 levels released from the platelets stimulated by 10 µM TRAP. Although the area under the curve (AUC) of small aggregates (9–25 µm) induced by 10 µM TRAP showed no significant correlation with the released HSP27 levels, AUC of medium aggregates (25–50 µm), large aggregates (50–70 µm) and light transmittance were significantly correlated with the released HSP27 levels. TRAP-induced phosphorylation of HSP27 was truly suppressed by deguelin, an inhibitor of Akt, in the platelets from a healthy subject. These results strongly suggest that TRAP-induced activation of Akt in addition to p38 MAP kinase positively regulates the release of phosphorylated-HSP27 from human platelets, which is closely related to the platelet hyper-aggregation in type 2 DM patients.

## 1. Introduction

Atherothrombosis, which is triggered by activated human platelets and caused high mortality or disturbance of daily life, is a major health concern to contribute cerebrovascular events and cardiovascular diseases [1,2]. Activated human platelets tethering at the sites of vascular injury responsively secrete autocrine/paracrine mediators such as adenosine diphosphate (ADP), release thromboxane A2 and promote repairment of vascular injury [1]. In addition, as granule contents, mitogenic mediators such as platelet-derived growth factor-AB (PDGF-AB) are secreted from activated platelets, which modify vascular endothelial/smooth muscle cell function resulting in the development of atherosclerosis [1]. Thrombin, a serine protease rapidly generated at the vascular injury sites from circulating prothrombin, is the most potent activator of platelets [1]. The responses of platelets to thrombin are mediated through the activation of protease-activated receptors (PARs) by the thrombin-mediated cleavage of the N-terminal exodomain [1]. It is currently known that two classes of PARs, PAR1 and PAR4, are expressed on human platelets, and PAR1 mainly contributes to their activation [1]. A 14-amino acid peptide of the new amino-terminus derived from the thrombin-mediated cleavage is revealed to be a potent thrombin receptor activator and is called thrombin receptor-activating protein (TRAP) [3]. Regarding the intracellular signaling of thrombin, it is generally recognized that several molecules including mitogen-activated protein (MAP) kinases and Akt/PKB play roles in the PAR-mediating platelet activation [4].

Type 2 diabetes mellitus (DM) is currently considered a major health problem not only in developed countries but also in developing ones [5]. Even in early stages of the disease, spontaneous platelet aggregation associated with the progression of atherosclerotic change is susceptible to development, so the patients possess an increase of lifetime risk to vascular complications such as cardiovascular diseases once they are diagnosed with DM [6]. Thus, it is widely established that the adequate control of platelet aggregation based upon the pathogenesis of platelet functions in DM is essential for the improvement of their prognosis. As for the functional changes of platelets in the disease, we have reported that a low-dose (1 µM) of ADP induces irreversible platelet microaggregation, namely the platelet hypersensitivity is observed, in type 2 DM patients [7]. We have also shown that the activation of p44/p42 MAP kinase and p38 MAP kinase are closely correlated with the hyper-aggregation in the collagen-stimulated platelets from type 2 DM patients [8]. However, the exact mechanism underlying platelet hyper-aggregation in DM patients remains to be clarified.

Heat shock proteins (HSPs), which could be expressed by a variety of biological stresses in mammalian cells, generally act as molecular chaperones that protect unfolded proteins from aggregation [9]. Heat shock protein 27 (HSP27) (HSPB1) is grouped into low-molecular-weight HSPs (HSPB), which exert multiple pleiotropic functions such as an anti-apoptotic factor [10,11], and a stabilizer of actin and microtubules of cytoskeleton in addition to molecular chaperones [9,12]. On the other hand, post-translational modifications such as phosphorylation could modulate the HSP27 functions [9]. Human HSP27 phosphorylations reportedly occur at three residues of serine (Ser-15, Ser-78 and Ser-82) [9,13]. In its resting state, HSP27 exists in an unphosphorylated aggregated form, whereas HSP27 is rapidly dissociated into dimers or monomers accompanied with the phosphorylation, which is speculated to be critical for its substrate binding and for showing specific functions [9,14]. It has been reported that thrombin induces the phosphorylation of HSP27 in human platelets [15]. It is generally known that HSP27 phosphorylation is catalyzed by members of the MAP kinase superfamily [9]. In the process of human platelet activation, it has been reported that collagen induces HSP27 phosphorylation through p38 MAP kinase [16]. We have shown that collagen-induced HSP27 phosphorylation is positively regulated by Rac, a low-molecular weight GTP-binding protein, via p44/p42 MAP kinase in human platelets, leading to PDGF-AB secretion [17]. Furthermore, we have recently reported that, in type 2 DM patients, phosphorylated-HSP27 stimulated by collagen in their platelets is released in association with the hyper-aggregation [18]. However, the clinical relevance of the phosphorylation of HSP27 in human platelets is not sufficiently understood.

In the present study, we conducted the investigation into the relationship between the phosphorylation of HSP27 and TRAP-stimulated platelet activation in the patients of type 2 DM. We herein show that TRAP-induced activation of Akt in addition to p38 MAP kinase is involved in the release of phosphorylated-HSP27 from human platelets, which is correlated with the acceleration of platelet aggregation in type 2 DM patients.

## 2. Results

### 2.1. Characterization of the Subjects

The characteristics of the subjects (*n* = 47) are presented in Table 1. Among them, 19 patients were adopted for the analysis of Western blot. The HbA1c levels of the subjects for Western blot analysis and enzyme-linked immunosorbent assay (ELISA) (Enzo Life Science, Inc., Plymouth Meeting, PA, USA) were 8.4% ± 1.2% and 8.5% ± 1.3%, respectively, and those were significantly greater than the upper limit of normal range (5.9%). The anthropometric indexes ranged within the normal limits of Japanese individuals, and the significant changes of metabolic markers were not observed.

### 2.2. Platelet Aggregation and the Phosphorylation of Heat Shock Protein 27 (HSP27), Akt, p44/p42 Mitogen-Activated Protein (MAP) Kinase and p38 MAP Kinase in the Subjects

**Figure 1:**
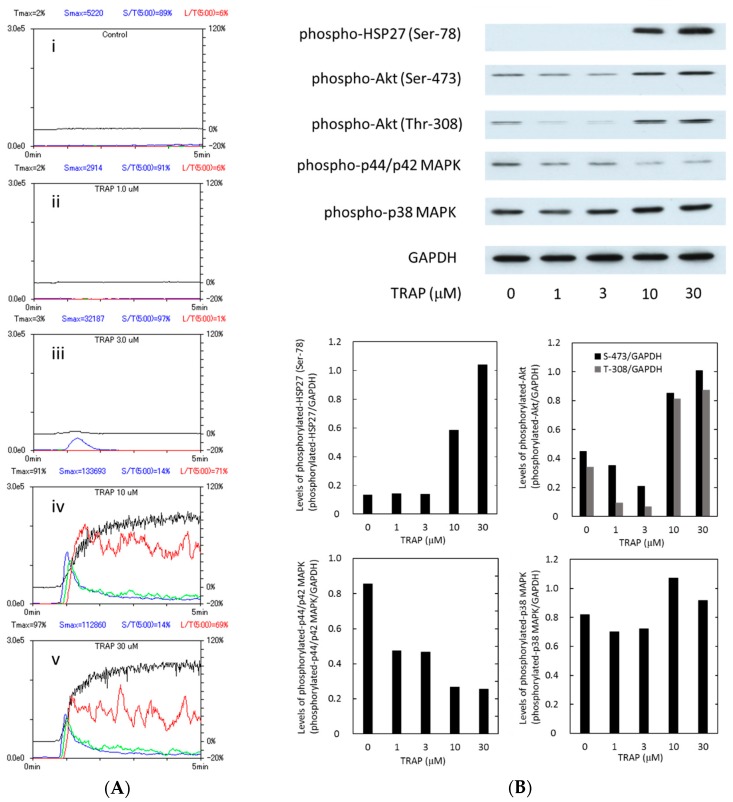
Representative patterns of platelet aggregation induced by thrombin receptor-activating protein (TRAP) as detected by an aggregometer using laser-scattering system and representative data showing the TRAP-induced HSP27 phosphorylation in platelets from type 2 diabetes mellitus (DM) patients. Platelet-rich plasma (PRP) from type 2 DM patients was stimulated by TRAP (0, 1.0, 3.0, 10 and 30 µM) in an aggregometer at 37 °C for 4 min with a stirring speed of 800 rpm. (**A**) time-dependent changes in the platelet aggregation after stimulation by 0 µM (i); 1.0 µM (ii); 3.0 µM (iii); 10 µM (iv) and 30 µM (v) are shown. The black line indicates the percentage of transmittance of each sample (the isolated platelets were recorded as 0%, and platelet free plasma was recorded as 100%). The blue line indicates small aggregates (9–25 µM); green line, medium aggregates (25–50 µm); red line, large aggregates (50–70 µM); (**B**) the reaction was terminated by the addition of an ice-cold EDTA (10 mM) solution. The lysates of platelets were subjected to a Western blot analysis making use of antibodies against phospho-specific HSP27 (Ser-78 and Ser-82), phospho-specific Akt (Ser-473 and Thr-308), phospho-specific p44/p42 mitogen-activated protein (MAP) kinase, phospho-specific p38 MAP kinase and GAPDH. The bands were quantified using the ImageJ software program (National Institutes of Health, Bethesda, MD, USA) as the counts of pixels. The bands of phospho-HSP27, phospho-Akt, phospho-p44/p42 MAP kinase and phospho-p38 MAP kinase were normalized to the GAPDH bands. The ratio is presented for each value. The **upper** panel indicates the results of a Western blot analysis, and **lower** panels indicate each ratio.

Representative patterns of TRAP-induced platelet aggregation in type 2 DM patients in the study groups analyzed by an aggregometer (PA-200 apparatus, Kowa Co., Ltd., Tokyo, Japan) using the laser scattering system are shown in Figure 1A. TRAP-induced platelet aggregation by doses more than 10 µM. In that case, the large platelet aggregate (50–70 µm) was markedly induced by 10 µM TRAP, and the ratio reached 71% of total aggregates.

Human HSP27 is phosphorylated at three residues of serine (Ser-15, Ser-78 and Ser-82) [9,13]. In our previous study [18], we have demonstrated that the levels of HSP27 phosphorylation at Ser-78 by collagen are significantly correlated with the levels of released phosphorylated-HSP27 in human platelets. Therefore, we examined the effects of TRAP on the phosphorylation of HSP27 (Ser-78). TRAP remarkably stimulated the phosphorylation of HSP27 (Ser-78) over the range between 10 and 30 µM (Figure 1B). The levels of phosphorylated-HSP27 seem to be directly proportional to the platelet aggregation.

It has recently been reported that the 3-phosphoinositide-dependent protein kinase-1 (PDK1)-regulating phosphorylation of Akt is involved in thrombin-induced platelet activation [19]. We have previously reported that p44/p42 MAP kinase and p38 MAP kinase participate in the ADP-induced phosphorylation of HSP27 in human platelets [20]. Therefore, we examined whether TRAP elicits the activation of Akt, p44/p42 MAP kinase or p38 MAP kinase in the platelets derived from type 2 DM patients. TRAP markedly induced the phosphorylation of Akt (Ser-473 and Thr-308) and p38 MAP kinase (Figure 1B). However, the phosphorylation of p44/p42 MAP kinase seems to be induced but suppressed by TRAP (Figure 1B).

### 2.3. The Relationship between Levels of Phosphorylated-HSP27 and Those of Phosphorylated-Akt (Thr-308) Induced by TRAP in the Platelets of Type 2 Diabetes Mellitus (DM) Patients

**Figure 2:**
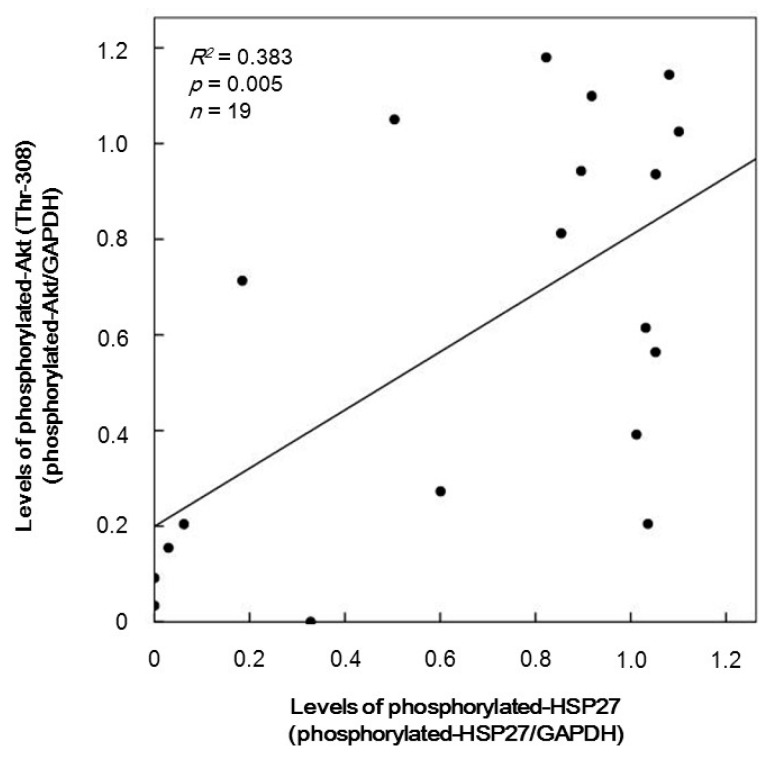
The relationship between individual levels of the phosphorylation of HSP27 (Ser-78) and Akt (Thr-308) induced by TRAP in platelets from type 2 DM patients. The baseline levels in unstimulated samples were subtracted from each level of the individual HSP27 phosphorylation and Akt phosphorylation stimulated by 10 µM of TRAP for 4 min, and the net changes are presented as levels of phosphorylated-HSP27 (Ser-78) and phosphorylated-Akt (Thr-308), respectively. Each of the data were determined by a Western blot analysis using the ImageJ software program. These data were plotted and analyzed by linear regression analysis.

In order to investigate the relationship between HSP27 phosphorylation and Akt activation in the TRAP-stimulated platelets, we plotted the individual levels of phosphorylated-HSP27 against those of phosphorylated-Akt induced by 10 µM TRAP in the group of Western blotting. The levels of phosphorylated-Akt (Thr-308) were significantly correlated with those of the HSP27 phosphorylation (Ser-78) (*R*^2^ = 0.383, *p* = 0.005) (Figure 2).

### 2.4. The Relationship between Levels of Phosphorylated-HSP27 and Those of Phosphorylated-p38 MAP Kinase Induced by TRAP in the Platelets from Type 2 DM Patients

**Figure 3:**
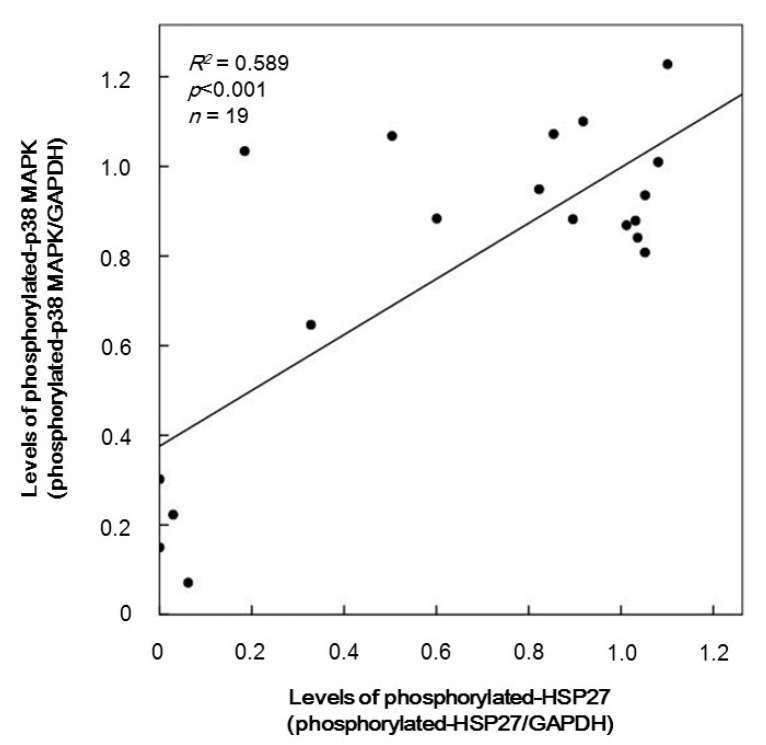
The relationship between individual levels of the phosphorylation of HSP27 (Ser-78) and p38 MAP kinase induced by TRAP in platelets from type 2 DM patients. The baseline levels in unstimulated samples were subtracted from each level of the individual HSP27 phosphorylation and p38 MAP kinase phosphorylation stimulated by 10 µM of TRAP for 4 min, and the net changes are presented as levels of phosphorylated-HSP27 (Ser-78) and phosphorylated-p38 MAP kinase, respectively. Each of the data were determined by a Western blot analysis using the ImageJ software program. These data were plotted and analyzed by linear regression analysis.

It is generally recognized that HSP27 is phosphorylated via p38 MAP kinase activation [9]. We next plotted the individual levels of phosphorylated-HSP27 against those of phosphorylated-p38 MAP kinase induced by 10 µM TRAP in the group of Western blotting. The phosphorylated levels of p38 MAP kinase were directly proportional to the phosphorylated-HSP27 levels (Ser-78) (*R*^2^ = 0.589, *p* < 0.001) (Figure 3).

### 2.5. The Relationship between Levels of HSP27 Phosphorylation and Levels of Released Phosphorylated-HSP27 Induced by TRAP in the Platelets of Type 2 DM Patients

**Figure 4:**
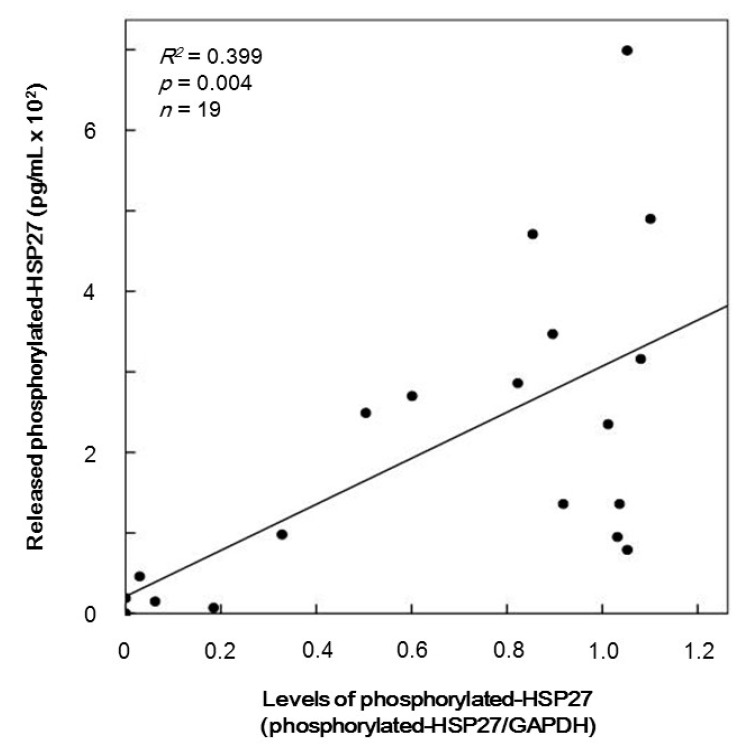
The relationship between individual levels of HSP27 phosphorylation (Ser-78) in the platelets and the release of phosphorylated-HSP27 levels induced by TRAP in type 2 DM patients. The baseline levels without stimulation were subtracted from each of the individual HSP27 phosphorylation levels, and the release of phosphorylated-HSP27 levels in the conditioned plasma after platelet aggregation stimulated by 10 µM of TRAP for 4 min were collected. Each of the data regarding the phosphorylation of HSP27 in platelets and the levels of release of phosphorylated-HSP27 were determined by a Western blot analysis using the ImageJ and enzyme-linked immunosorbent assay (ELISA), respectively. These data were plotted and analyzed by linear regression analysis.

We have recently reported that HSP27 is released from the platelets stimulated by collagen of type 2 DM patients accompanied with its phosphorylation [18]. Thus, we plotted the individual levels of released phosphorylated-HSP27 against those of phosphorylated-HSP27 induced by 10 µM TRAP in the group for Western blotting. The levels of the release of phosphorylated-HSP27 were significantly correlated with those of phosphorylated-HSP27 (Ser-78) (*R*^2^ = 0.399, *p* = 0.004, *n* = 16) (Figure 4).

### 2.6. The Relationship between Levels of Secreted Platelet-Derived Growth Factor-AB (PDGF-AB) and Levels of Release of Phosphorylated-HSP27 Induced by TRAP from the Platelets of Type 2 DM Patients

**Figure 5:**
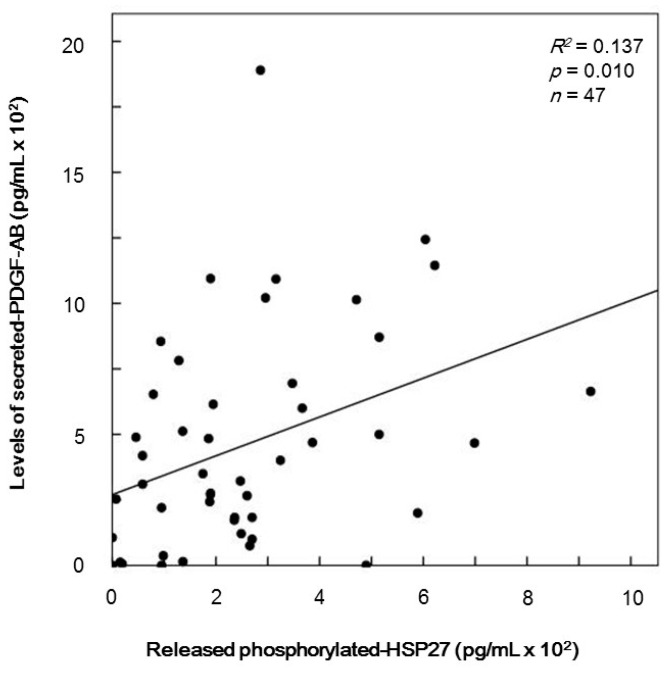
The relationship between individual levels of released phosphorylated-HSP27 and the levels of secreted platelet-derived growth factor-AB (PDGF-AB) in the supernatant of the conditioned plasma after platelet aggregation stimulated by 10 µM of TRAP for 4 min were determined using specific ELISA kits. The baseline levels without stimulation were subtracted from each of the individual levels of the release of phosphorylated-HSP27 and PDGF-AB. These data were plotted and then analyzed by linear regression analysis.

PDGF-AB that is secreted accompanied with platelet activation promotes the progress of atherosclerosis as a mitogenic growth factor [1]. We have recently demonstrated that the levels of released phosphorylated-HSP27 induced by collagen are significantly correlated with secreted PDGF-AB levels from the platelets of type 2 DM patients [18]. Thus, we compared the TRAP (10 µM)-induced PDGF-AB secretion with the individual levels of release of phosphorylated-HSP27 from the platelets of type 2 DM patients. The levels of secreted PDGF-AB were closely related with the levels of release of phosphorylated-HSP27 (*R*^2^ = 0.137, *p* = 0.010, *n* = 47) (Figure 5).

### 2.7. The Relationship between Parameters for Platelet Aggregation and the Levels of Release of Phosphorylated-HSP27 Induced by TRAP in Type 2 DM Patients

**Figure 6:**
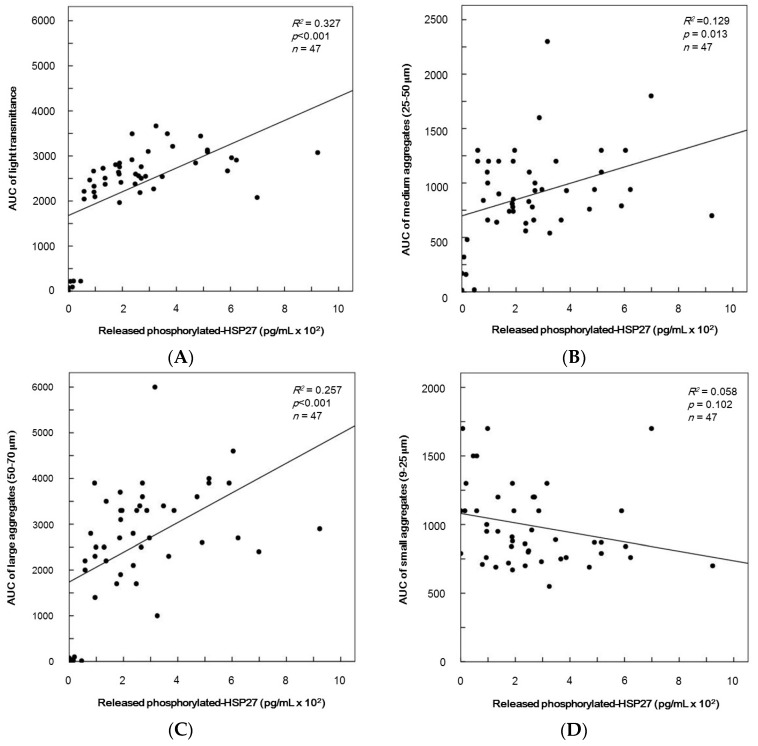
The relationship between individual levels of release of phosphorylated-HSP27 and the area under the curve (AUC) of platelet aggregation induced by TRAP in type 2 DM patients. The levels of release of phosphorylated-HSP27 in the supernatant of the conditioned plasma after platelet aggregation stimulated by 10 µM of TRAP for 4 min was determined using a specific ELISA kit. AUC of platelet aggregation stimulated by 10 µM of TRAP for 4 min were determined by an aggregometer (PA-200 apparatus, Kowa Co., Ltd., Tokyo, Japan) with laser-scattering system recorded individually by the size of aggregates, (**A**) AUC of total transmission; (**B**) AUC of medium aggregates; (**C**) AUC of large aggregates; (**D**) AUC of small aggregates. These data were plotted and then analyzed by linear regression analysis.

To clarify whether TRAP-induced aggregation of platelet is related to the release of phosphorylated-HSP27 from the activated platelets, we plotted the levels of individual area under the curve (AUC) of platelet aggregation including transmittance and the size of aggregates against the levels of released phosphorylated-HSP27 induced by 10 µM TRAP in the platelets of type 2 DM patients. The levels of released phosphorylated-HSP27 were significant to AUC of light transmittance (Figure 6A). In addition, AUC of medium aggregates (25–50 µm) and large aggregates (50–70 µm) were directly proportional to the levels of released phosphorylated-HSP27 (*R*^2^ = 0.129, *p* = 0.013; *R*^2^ = 0.257, *p* < 0.001; *R*^2^ = 0.327, *p* < 0.001, respectively, *n* = 47) (Figure 6B,C). However, there was no significant relationship between AUC of small aggregates (9–25 µm) and the levels of extracellular phosphorylated-HSP27 (*R*^2^ = 0.058, *p* = 0.102, *n* = 47) (Figure 6D).

### 2.8. The Effect of Deguelin on the TRAP-Induced Phosphorylation of HSP27 in Human Platelets

**Figure 7:**
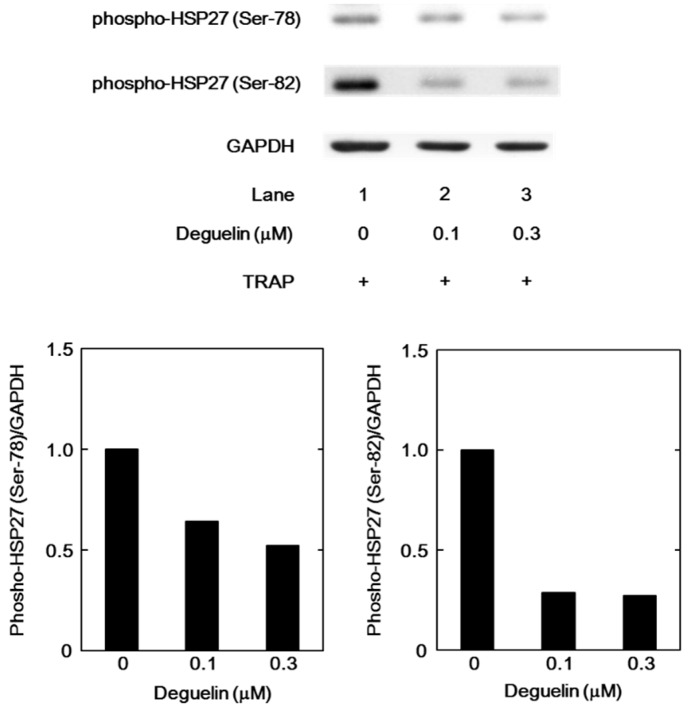
The effect of degueline on the TRAP-induced phosphorylation of HSP27 in the platelets from a healthy subject. PRP from a healthy subject was pretreated with various doses of deguelin for 15 min and then stimulated by 20 µM of TRAP at 37 °C for 4 min. The reaction was terminated by the addition of an ice-cold EDTA (10 mM) solution. The lysates of platelets were subjected to a Western blot analysis making use of antibodies against phospho-specific HSP27 (Ser-78 and Ser-82) and GAPDH. The bands were quantified by ImageJ as the counts of pixels. The bands of phospho-HSP27 were normalized to the GAPDH bands. The ratio is presented for each value. The upper panel indicates the bands of Western blot, and lower panels indicate each ratio.

We further examined the effect of deguelin, an inhibitor of Akt [21], on the TRAP-induced phosphorylation of HSP27 in the platelets derived from a healthy donor. Deguelin markedly reduced the TRAP-induced phosphorylation of HSP27 at Ser-78 and Ser-82 in a dose-dependent manner in the range between 0.1 and 0.3 µM (Figure 7).

## 3. Discussion

We have recently shown that in type 2 DM patients the release of HSP27 from platelets is accompanied with its phosphorylation induced by collagen, which is correlated with the acceleration of platelet aggregation [18]. Thrombin is generally recognized to be one of the potent stimulators of platelet aggregation [3,4]. It has been shown that HSP27 is phosphorylated in thrombin-activated human platelets [15]. In the present study, we investigated whether thrombin receptor-mediating signaling by TRAP could provoke the HSP27 release from the platelets in type 2 DM patients. We found that TRAP (10 or 30 µM) induced the phosphorylation of HSP27 at Ser-78, which seems to be concomitant with the platelet aggregation. It is recognized that MAP kinases and Akt/PKB are involved in the intracellular signaling of thrombin-activated PAR1 [4]. In aortic smooth muscle cells, we have previously reported that Akt acts as a potent regulator in thrombin-induced HSP27 phosphorylation [22]. To investigate the mechanism underlying the TRAP-induced phosphorylation of HSP27, we examined the effects of TRAP on the phosphorylation of Akt, p44/p42 MAP kinase and p38 MAP kinase in the platelets from type 2 DM subjects. We found that the phosphorylation of Akt (Ser-473 and Thr-308) and p38 MAP kinase, but not p44/p42 MAP kinase, were elicited by TRAP. It seems unlikely that p44/p42 MAP kinase is involved in the TRAP-induced HSP27 phosphorylation in the platelets from type 2 DM patients. Thus, we next analyzed the relationship between the levels of phosphorylated-HSP27 (Ser-78) and the levels of phosphorylated-Akt (Thr-308) or phosphorylated-p38 MAP kinase individually in type 2 DM patients. We showed that the TRAP-induced levels of phosphorylated-HSP27 were significantly correlated with those of phosphorylated-Akt (Thr-308) or phosphorylated-p38 MAP kinase. It is well known that p38 MAP kinase is activated by phosphorylation of serine and threonine residue by dual specificity MAP kinase kinase [23], and that Akt is activated by the phosphorylation of Thr-308 by phosphatidylinositol 3-kinase-dependent PDK1 [21]. Based on these findings, it seems likely that TRAP elicits HSP27 phosphorylation through the activation of Akt as well as p38 MAP kinase in the platelets derived from type 2 DM patients. To clarify whether TRAP-induced activation of Akt is involved in the HSP27 phosphorylation in human platelets, we examined the effect of deguelin [24] on the TRAP-induced phosphorylation of HSP27. We found that the TRAP-induced phosphorylation of HSP27 (Ser-78 and Ser-82) was truly suppressed by deguelin in the platelets. However, the suppression by deguelin was not complete but partial. Thus, it is likely that p38 MAP kinase also plays a role in the TRAP-induced HSP27 phosphorylation in addition to Akt in human platelets. Taking our present results into account, it is probable that Akt in addition to p38 MAP kinase is involved in the phosphorylation of HSP27 induced by TRAP in the platelets from type 2 DM patients. Regarding the present results of Western blot image shown in Figure 1B, it seems that Akt (Ser-473 and Thr-308), p44/p42 MAP kinase and p38 MAP kinase are phosphorylated even in the absence of TRAP stimulation. It is likely that these kinases are constitutively activated without any stimulation in the platelets from type 2 DM patients. On the other hand, it seems that the levels of Akt phosphorylation stimulated by 1 and 3 µM TRAP are lower than those without TRAP. It is probably due to the differences of sample conditions for Western blotting because of the lack of reproducibility through the present experimental series.

We next investigated whether the phosphorylated-HSP27 induced by TRAP is released from the platelets of type 2 DM patients. We measured the release of phosphorylated-HSP27 under the stimulation of 10 µM TRAP and analyzed the relationship between the individual levels of extracellular phosphorylated-HSP27 and intracellular TRAP-induced phosphorylation of HSP27 in the platelets. The levels of released phosphorylated-HSP27 stimulated by TRAP were directly proportional to the levels of intracellular phosphorylated-HSP27. Therefore, our findings suggest that the TRAP-induced phosphorylation of HSP27 elicits the release of HSP27 from the TRAP-activated platelets into circulation.

We demonstrated here that there is a close correlation between the individual levels of the TRAP-induced release of phosphorylated-HSP27 and the secretion of PDGF-AB, a mitogenic mediator promoting atherosclerosis [1], from the platelets derived from type 2 DM patients. In addition, the levels of released phosphorylated-HSP27 induced by TRAP were significantly correlated with the AUC of light transmittance in the platelet aggregation in type 2 DM patients. Regarding the size of platelet aggregates, AUC of medium aggregates (25–50 µM) and that of large aggregates (50–70 µM) was directly proportional, whereas the AUC of small aggregates (9–25 µM) was not significant (*p* = 0.058) but looks inversely proportional. Taking our findings into account, it is probable that the phosphorylated-HSP27 release reflects the hyper-aggregation of platelets in the pathological state of type 2 DM. Evidence is accumulating that HSP27 not only intracellularly acts as a molecular chaperone but also extracellularly affects the behavior of the surrounding environment [9]. It has been reported that HSP27 released from macrophages protectively acts against the development of atherosclerosis [25], whereas extracellular HSP27 mediates angiogenesis by increasing vascular endothelial growth factor transcription in vascular endothelial cells [26]. As for immunity, another report has proposed that HSP27 upregulates pro-inflammatory factors including interleukin (IL)-1β via the activation of nuclear factor-κB as well as anti-inflammatory factors such as IL-10 in macrophages [27]. It has recently been shown that HSP27 is released from myocardium after global ischemia in humans, and that HSP27 elicits a pro-inflammatory effect through toll-like receptors in mouse coronary vascular endothelial cells [28]. It is well recognized that platelet activation is provoked by the initial tethering at the injured vascular sites, exposing a subendothelial collagen [1,2]. In addition, thrombin is rapidly generated at the vascular injury sites from circulating prothrombin, resulting in the amplification of platelet aggregation [1]. We have recently reported that collagen induces the release of phosphorylated-HSP27 from the platelets of type 2 DM patients [18]. Based on these findings, it is likely that collagen and thrombin cooperatively activate platelets in type 2 DM patients, resulting in the modulation of the pathogenesis of atherosclerosis through HSP27 release accompanied with PDGF-AB secretion. The phosphorylation of HSP27 in the platelets might propose a possible therapeutic aspect or a novel tool for the evaluation of pathologically accelerated platelet aggregation. It has recently been reported that the ratio of non-responders to clopidogrel, a P2Y12 antagonist, in the subjects with DM or impaired glucose tolerance is greater than the subjects without them in the cohort consisting of patients with minor ischemic stroke or transient ischemic attack [29]. In addition, the non-responders have a tendency towards higher aggregation induced by TRAP [29]. Taking the previous report into account as a whole, it is possible that the TRAP-activating Akt involvement in the release of phosphorylated-HSP27 from platelets as shown here is significant in the patients with DM. Further investigation is necessary to elucidate the details underlying released phosphorylated-HSP27 from human platelets.

## 4. Materials and Methods

### 4.1. Materials

TRAP (H-Ser-Phe-Leu-Leu-Arg-Asn-Pro-Asn-Asp-Lys-Tyr-Glu-Pro-Phe-OH trifluoroacetate salt) was purchased from Bachem AG (Budendorf, Switzerland). Phospho-specific HSP27 (Ser-78) antibodies were purchased from Stressgen Biotechnologies (Victoria, BC, Canada). Phospho-specific Akt (Ser-473 and Thr-308) antibodies, phospho-specific p44/p42 MAP kinase antibodies, phospho-specific p38 MAP kinase antibodies and phospho-specific HSP27 (Ser-82) antibodies were purchased from Cell Signaling Technology, Inc. (Beverly, MA, USA). GAPDH antibodies and deguelin were purchased from Santa Cruz Biotechnology, Inc. (Santa Cruz, CA, USA). The PDGF-AB ELISA kit was purchased from R&D (Minneapolis, MN, USA). The phosphorylated-HSP27 ELISA kit was purchased from Enzo Life Science, Inc. (Plymouth Meeting, PA, USA). Other materials and chemicals were obtained from commercial sources. Deguelin was dissolved in dimethyl sulfoxide. The maximum concentration of dimethyl sulfoxide was 0.1%, which did not affect the detection of protein expression by a Western blot analysis.

### 4.2. Subjects

The inclusion criteria for the study were the presence of type 2 DM according to the criteria of the World Health Organization. We excluded the patients who were complicated with a malignancy, infectious diseases including hepatitis B and hepatitis C, or autoimmune disorders. All participants were advised to avoid sleep deprivation or blood donation. The study was approved by the committee of the conduct of human research at National Center for Geriatrics and Gerontology (Obu, Japan), and at Gifu University Graduate School of Medicine (Gifu, Japam). Written informed consent was obtained from all of the patients and healthy donors.

### 4.3. Blood Sampling

Ten ml of blood was drawn from the vein between 8:00 and 9:00 after at least 15 min of bed rest to preserve steady state conditions. Sodium citrate (14 µM) was added to the blood immediately as an anticoagulant, and platelet-rich plasma (PRP) was obtained by centrifugation at 155× *g* for 12 min at room temperature. Platelet-poor plasma (PPP) was prepared from the residual blood by centrifugation at 1400× *g* for 5 min.

### 4.4. Platelet Aggregation

Platelet aggregation was measured using an aggregometer (PA-200 apparatus, Kowa Co., Ltd., Tokyo, Japan) with a laser-scattering system as described previously [7,8]. In brief, PRP was preincubated at 37 °C for 1 min with a stirring speed of 800 rpm. Platelet aggregation was monitored for 4 min after the addition of various doses of TRAP (0, 1, 3, 10 and 30 µM). When indicated, PRP was pretreated with various doses of deguelin for 15 min, and then the platelet aggregation was monitored for 4 min after the addition of 20 µM of TRAP or vehicle. The percentage of transmittance of the isolated platelets was recorded as 0%, and that of the appropriate PPP (blank) was recorded as 100%. Platelet aggregation was then terminated by the addition of ice-cold EDTA (10 mM). The conditioned plasma was collected and centrifuged at 10,000× *g* at 4 °C for 2 min. The supernatant was collected and stored at −80 °C. The pellet was washed twice with PBS, and then lysed immediately by boiling in a lysis buffer containing 62.5 mM Tris-HCl, pH 6.8, 2% sodium dodecyl sulfate (SDS), 50 mM dithiothreitol and 10% glycerol for a Western blot analysis.

### 4.5. Western Blot Analysis

A Western blot analysis was performed as described previously [30]. In brief, SDS-polyacrylamide gel electrophoresis (PAGE) was performed by the method described by Laemmli [31] in a 12.5% polyacrylamide gel. The proteins fractioned in the gels were transferred onto polyvinylidene fluoride (PVDF) membranes, and then the membranes were blocked with 5% fat-free dry milk in Tris-buffered saline with 0.1% Tween-20 (TBS-T, 20 mM Tris, pH 7.6, 137 mM NaCl, 0.1% Tween-20) for 2 h before incubation with the indicated primary antibodies. Peroxidase-labeled antibodies raised in a goat against rabbit IgG (KPL, Gaithersburg, MD, USA) were used as the secondary antibodies. The primary and secondary antibodies were diluted to their optimal concentrations with 5% fat-free dry milk in TBS-T. The peroxidase activity on the PVDF membrane was visualized with X-ray film by means of an ECL Western blotting detection system (GE Healthcare, Buckinghamshire, UK) following the manufacturer’s protocol. The bands were analyzed by densitometry using the ImageJ software program (National Institutes of Health, Bethesda, MD, USA). The quantitative data of each sample were obtained as the counts of pixels.

### 4.6. ELISA for PDGF-AB and Phosphorylated-HSP27

The levels of PDGF-AB and phosphorylated-HSP27 in the supernatant of the conditioned mixture after platelet aggregation were determined using ELISA kits for PDGF-AB and phosphorylated-HSP27, respectively, according to manufacture’s instructions.

### 4.7. Statistical Analysis

The data were presented as the mean ± SD. The statistical significance of the correlation between two variables, linear regression analysis was adopted using SPSS ver. 19.0 (IBM Japan Ltd., Tokyo, Japan) as the software. A probability of less than 5% was considered to be statistically significant.

## 5. Conclusions

In conclusion, our present findings strongly suggest that TRAP-induced activation of Akt in addition to p38 MAP kinase positively regulates the release of phosphorylated-HSP27 from human platelets, which is closely related to the acceleration of platelet aggregation in type 2 DM patients.

## Figures and Tables

**Table 1:** Characteristics of the study subjects.

Parameters	For Western Blotting	For ELISA
Total number	19	47
Gender (F/M)	(12/7)	(22/25)
Age (years)	71.5 ± 4.9	72.0 ± 6.6
DM duration (years)	15.1 ± 8.3	14.1 ± 7.6
Height (cm)	157.3 ± 7.7	157.1 ± 9.8
Weight (kg)	59.9 ± 8.5	59.5 ± 12.8
BMI	24.1 ± 2.7	23.9 ± 3.5
sBP (mmHg)	115.2 ± 17.3	119.8 ± 18.9
dBP (mmHg)	65.3 ± 11.5	67.1 ± 11.3
HbA1c (%)	8.4 ± 1.2	8.5 ± 1.3
Glu (mg/dL)	167.0 ± 57.5	165.3 ± 56.0
TC (mg/dL)	189.4 ± 42.2	184.1 ± 37.9
TG (mg/dL)	146.1 ± 109.3	130.3 ± 77.7
HDL (mg/dL)	49.0 ± 16.6	49.5 ± 13.7
Plt (×10^4^/µL)	19.1 ± 4.3	21.0 ± 4.9

ELISA indicates enzyme-linked immunosorbent assay; F, female; M, male; BMI, body mass index; sBP, systolic blood pressure; dBP, diastolic blood pressure; HbA1c, hemoglobin A1c; Glu, plasma glucose; TC, total cholesterol; TG, triglyceride; HDL, high-density lipoprotein; Plt, platelet counts. The data are presented as the means ± SD.

## Abbreviations

HSP27	heat shock protein 27
MAP	mitogen-activated protein
TRAP	thrombin-activating protein
DM	diabetes mellitus
AUC	area under the curve
GP	glycoprotein
ADP	adenosine diphosphate
PDGF-AB	platelet-derived growth factor-AB
PAR	protease-activated receptor
PDK1	3-phosphoinositide-dependent protein kinase-1
PRP	platelet-rich plasma
PPP	platelet-poor plasma
PAGE	polyacrylamide gel electrophoresis
PVDF	polyvinylidene fluoride
